# Evaluation of the efficacy of an appeasing pheromone diffuser product vs placebo for management of feline aggression in multi-cat households: a pilot study

**DOI:** 10.1177/1098612X18774437

**Published:** 2018-05-14

**Authors:** Theresa L DePorter, David L Bledsoe, Alexandra Beck, Elodie Ollivier

**Affiliations:** 1 Department of Behavioral Medicine, Oakland Veterinary Referral Services, Bloomfield Hills, MI, USA; 2 Qualitas BioSciences, LLC, Peoria, AZ, USA; 3 Ceva Santé Animale, Libourne, France

## Abstract

**Objectives:**

Aggression and social tension among housemate cats is common and puts cats at risk of injury or relinquishment. The aim of this study was to evaluate the effectiveness of a new pheromone product in reducing aggression between housemate cats.

**Methods:**

A new pheromone product (Feliway Friends) containing a proprietary cat-appeasing pheromone was evaluated for efficacy in reducing aggression between housemate cats via a randomized, double-blind, placebo-controlled pilot trial of 45 multi-cat households (pheromone [n = 20], placebo [n = 25]) reporting aggression for at least 2 weeks. Each household had 2–5 cats. Participants attended an educational training meeting on day (D) –7 and the veterinary behaviorist described behaviors to be monitored for 7 weeks using the Oakland Feline Social Interaction Scale (OFSIS), which assessed the frequency and intensity of 12 representative aggressive interactions. Participants were also provided with instructions for handling aggressive events, including classical conditioning, redirection by positive reinforcement and not punishing or startling the cat for aggressive displays. Punishment techniques were strongly discouraged. Plug-in diffusers with the pheromone product or placebo were utilized from D0–D28. Participants completed a daily diary of aggressive events and weekly OFSIS assessments through to D42.

**Results:**

Evolution of the OFSIS–Aggression score according to treatment group in the full analysis set population revealed a significant effect on time and treatment group. The OFSIS–Aggression score decreased over time from D0–D28 in both groups (time factor *P* = 0.0001) with a significant difference in favor of the verum *P* = 0.06); similar results were found considering the D0–D42 period (time factor *P* = 0.0001 [D0] and *P* = 0.04 [D42]).

**Conclusions and relevance:**

The OFSIS provided a quantifiable measure of the frequency and intensity of 12 intercat interactions reflecting conflict between cats. The cat-appeasing pheromone is a promising treatment for the management of aggression between housemate cats in multi-cat households.

## Introduction

Domestic cats (*Felis catus*) have established themselves as a worldwide presence owing to their excellent predatory skills, and continue to expand their international popularity.^1–5^ Humans and domestic cats bond quite well and it is not uncommon for cat owners to open their homes to more than one cat. The domestic cat is currently the most common pet in many countries around the world. The total cat population in the USA is estimated to be 94.2 million (2017); in Europe it is 102.7 million cats (2016).^1,4^ Thirty percent of US households own at least one cat, with an average of 2.1 cats per cat-owning household (2012).^1^ More people own dogs than cats, but the number of cats is higher because cat owners tend to have two or more cats sharing their home.^1^

Domestic cats, like most other felids, are nocturnal, secretive, solitary survivors and skilled hunters. Cats are socially flexible and may be quite independent or live in close contact. Feline social interactions are influenced by many factors such as their early social experiences and the availability of resources. They are committed carnivores and driven by independent urges to stalk, catch or kill their prey. Such characteristics mean the domestic cat does not require coordinated hunting with other felids.^2,5,6^ While cats are capable of complex social behaviors and do form strong social bonds with familiar or related cats, they often do not eagerly accept an unfamiliar cat. Close affiliative socially bonded feline friendships are characterized by co-sleeping, nose touching and allogrooming – behaviors most often expressed by cats that have known each other since kittenhood.^2,5,6^

However, some cats are forced to live together without the ability to leave a social group created by their owners that is not to their liking. Aggression and social tension among housemate cats is common and puts cats at risk of injury or relinquishment.^7^ In such cases, they may display a wide range of overt or passive aggressive distance-increasing behaviors. Overt displays of aggression include growling, hissing, screaming, spitting, attacking, chasing and biting.^8^ Passive displays include staring, blocking and hiding. In order to avoid conflict some cats choose to run away, hide or spend more time avoiding direct interactions. Cats that live indoors may distribute themselves within the home or may even favor going outdoors. Cats that are outdoors have greater opportunity to disperse themselves. Once conflicts occur, cats may continue to exist in a tense social situation for months or years, which affects their daily welfare, increases stress and reduces freedom to access resources without fear of confrontation. Harmony in any household is desirable and a cat that does not get along with a housemate cat faces a greater risk of relinquishment, daily stresses and potential for injury. In addition, there are cats living in shelters that would be candidates to be added to homes if owners could be sure their cats would get along. The wellbeing of millions of cats may be enhanced if cats got along with housemate cats.

Domestic canids have well developed social relationships and the appeasement behaviors to maintain them with a minimum of conflict, whereas felids’ natural social groups and relationships are based on matrilineal bonds and preferred associates. Felids, from house cats to cheetahs, generally prefer to avoid other cats except when parenting or when sexually attracted.^8,9^ As cats are not dependent on social relationships to hunt cooperatively, they are not required to reconcile following agonistic interactions. They therefore almost uniformly lack appeasement and reconciliative behaviors.^8,9^ When cats are forced into involuntary interaction, they prefer to avoid direct physical conflict.^6,10^ The cost vs benefit ratio of such events is high and risk of injury great. However, lacking the methods of appeasement common in dogs (eg, averted gaze, rolling on the back, licking the face, submissive urination), feline intra-specific aggression may quickly escalate to a serious physical or vocal confrontation. According to a 2016 literature review by the American Veterinary Medical Association on the welfare implications of declawing of domestic cats, there was not strong evidence that declawing increases the risk of undesirable behaviors or decreases the observance of desirable behaviors.^11^ However, a recent telephone survey suggests a higher incidence of elimination misbehaviors by cats that are declawed.^12^ Moreover, a recent retrospective study also identified that onychectomy (declawing) could affect feline behavior, with significantly increased odds of aggression in declawed cats with radiographic evidence of residual P3 fragments, and even cats with no evidence of residual P3 had an increased odds of biting (odds ratio 3.0).^13^

Kittens will form strong social relationships during the ‘sensitive period’ or the window of optimal socialization that occurs between 2 and 7 weeks of age.^14^ Curiously, during these first few weeks of age is the time of natural cat-appeasing pheromone release by the queen; coincidentally or causally at a time when kittens are living in groups with very few conflicts and competition. When they are adults, however, cats may not invest significant energy in maintaining social bonds or reconciling broken bonds following conflict. Not all cats living within the same home may be members of the same social groups; there may be pairs or triads of cats that are affiliative and other individuals or dyads that are not.^10^

Intercat conflict may begin when cats are first introduced to one another, or it may develop among cats that formerly had good affiliative bonds. Conflict may develop abruptly following a specific incident (eg, the introduction of a new pet in the home, one cat returning from a veterinary visit or a change at home that may have had an impact on availability of resources such as food or elimination places). Conflict may also develop gradually. The conflict may be obvious if cats are hissing, swatting, growling or screaming, or quite subtle, such that passive avoidance may go unnoticed by even observant owners.

Conflict between housemate cats may have elements of fear, anxiety, self-defense or territorial defense. Other behavioral consequences may include urine retention, undesired elimination outside the litter box or urine marking. ^8,15–17^ Medical consequences may include feline idiopathic cystitis.^8,15^ Some cats may face restricted access to food owing to social conflicts and thus may gulp or overeat when they do eat, whereas other cats may suffer weight loss.^18^

Treatment of severe conflicts between housemate cats has been described as challenging and is often considered difficult to completely resolve.^8,15–17^ Reported therapies range from medications such as diazepam, clomipramine, fluoxetine and buspirone to complex reintroduction programs.^8,15–17^ While many cats that struggle with conflicts may benefit from medications, it may be difficult to administer oral medications and often owners are hesitant to ‘drug’ their cats. Management and behavior modification strategies are tedious, time consuming and challenging for owners to implement with the necessary consistency. Controlled reintroductions may occur while one or more cats are observed, crate-confined or harnessed. Such programs may be successful, but may also be detrimental if carried out improperly.^8,15–17^ Commonly recommended punishment strategies for aggressive displays have included blasting foghorns, spraying with water, yelling and clapping hands. All are more likely to startle the cats rather than facilitate a peaceful reconciliation.^8,15–17^ Punishment strategies do not create an atmosphere suitable for reconciliation and a single traumatic interaction may reverse weeks or months of meticulous behavior modification and can be extremely frustrating for owners and the cats.

Cats, like most mammals, are able to communicate with other members of the species by leaving and detecting messages in the form of pheromones deposited in the environment.^19–21^ Pheromones are substances secreted from sebaceous mucous or sweat glands by an individual and received by others of the same species, in which it releases a specific reaction.^19,20^ Pheromones are classified by their modes of action, rather than chemical composition. The chemical diversity of pheromones ranges from small, volatile molecules to sulfated steroids to large families of proteins.

Cats may deposit pheromones by leaving scents in the environment by means of face rubbing (eg, facial pheromones), urine marking or scratching on upright surfaces (eg, feline interdigital semiochemical), whereas appeasing pheromones are released from the mammary area.^19^ Pheromones are then received by the vomeronasal organ (VNO), a paired tubular structure located just above the hard palate near the intranasal septum.^19–21^ When pheromone molecules are first detected by a cat, further olfactory investigation occurs, which is characterized by a tongue lick to the nose, followed by the cat’s gazing in a ‘thoughtful’, ‘preoccupied’ fashion while the upper lips are raised slightly and fluffed, with the mouth slightly open.^22^ This is called a flehmen response or ‘gape’, which serves to facilitate gathering the pheromones into the passageways.^19,21,22^ The pheromone molecules then interact with receptors in the VNO, which stimulate structures within the limbic system to alter the animal’s emotional state or activate physiologic effects.

Feline-appeasing pheromones are released by mammary sebaceous glands of the queen during the nursing period, appearing 3–4 days after parturition and persisting until 2–5 days after the weaning of the kittens (2–3 months maximum).^19,20^ These pheromones are most biologically active during the socialization period and thus are likely to enhance bond formation while comforting and reassuring the neonates. A commercially available synthetic copy of the dog appeasing pheromone (Adaptil, previously known as DAP; Ceva Santé Animale) has demonstrated calming properties in a variety of clinically and behaviorally relevant situations.^23–31^ Similarly to the dog appeasing pheromone, a synthetic analog of the feline-appeasing pheromone has been developed, which is now commercially available (the formulation used in this trial is currently commercially available as Feliway MultiCat in the USA and as Feliway Friends in Europe; Ceva Santé Animale). One study demonstrated that maternal pheromones reduced aggressive interactions during a controlled exposure of dyads of adult cats.^32^ Case reports have suggested an influence on feline aggression between housemate cats.^33,34^ It is hypothesized that the addition of the synthetic pheromone to the environment in multi-cat households may reduce intercat aggression by enhancing social affiliations and increasing the cats’ sense of wellbeing. The present pilot study was intended to test this hypothesis.

## Materials and methods

A double-blinded, randomized, placebo-controlled field trial was conducted to evaluate the clinical effectiveness of a 28 day treatment period with a proprietary synthetic cat-appeasing pheromone analog (Feliway Friends) in the reduction of intercat aggression. The pheromone (2% w/w) solution and the equivalent placebo solution without the pheromone were provided with a pair of plug-in diffusers and a 50 ml flask containing 48 ml of the respective test materials. The test article and the placebo diffusers, flasks and solutions were identical in appearance.

The test unit was the individual household. Forty-five multi-cat households of 2–5 cats each were enrolled. Demographic information regarding the duration and nature of the aggression, nature of onset (abrupt vs gradual) and number of cats in the household was obtained. Each enrolled household was reported by the owner to have a history of intercat aggression of at least 2 weeks’ duration, as evidenced by at least one fight and four aggressive encounters occurring within the previous 2 weeks. Fights were described and defined by tense body tone, freezing, staring, agitated (angry) vocalizations and may have included a bite, scratch and physical contact such as swatting. Physical confrontations need not necessarily have resulted in injury. The cats’ aggressive encounters (fights or displays) could include any of the following signs: stalking, chasing, fleeing, hiding, swatting, staring, blocking, growling, hissing, spitting, crouching, tail twitching or tail puffing. Video examples were shown to the volunteers to help them distinguish between fight encounters and play behaviors. Play was described as being characterized by the cats’ loose muscle tone, more physical contact, including gentle wrapping of the paws, mock predatory pouncing, and inhibited mouthing and the lack of accompanying vocalizations characteristic of aggression.

Recruitment was a convenience sample of households in the Midwestern United States and potential participants were recruited by distribution of an information flyer describing the clinical trial. Social and broadcast media were also used to recruit potential cases, and flyers were distributed. Interested cat owners who believed they met the inclusion criteria contacted the behaviorist by telephone or via a study-dedicated email address. Owners were preliminarily screened by a checklist-directed telephone interview to determine eligibility.

**Figure 1:**
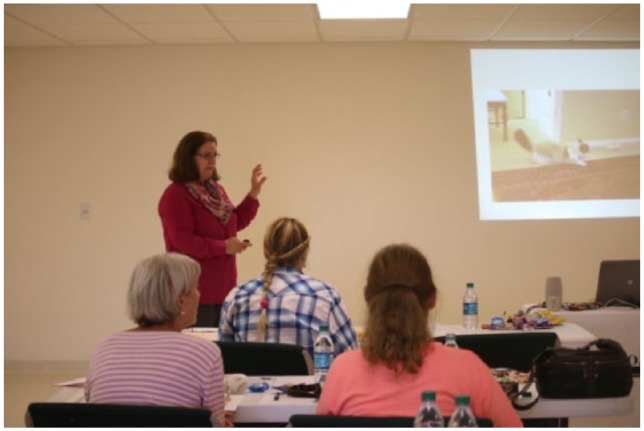
Volunteers attended an educational meeting at the time of enrollment

To complete enrollment in the study, owners were required to attend one of a series of scheduled meetings held at Oakland Veterinary Referral Services (Bloomfield Hills, MI, USA). Enrollment meetings consisted of a PowerPoint presentation on feline behavior and aggression, as well as the particulars of the study (Figure 1). As transport of cats to the hospital may have increased stress, influenced patterns of social interactions or caused general changes in activity, the owners were instructed not to bring their cats to the meeting and no cats were evaluated directly by the investigators during the study. Incentives for participants consisted of a $50 gift card upon completion of the study and a gift of the pheromone test product when it became commercially available.

All cats were at least 6 months of age at enrollment. No restrictions were placed on breed or sex, but sexually intact males were excluded. All cats spent at least 16 h a day indoors with unrestricted access to common areas and each other throughout the home. Cats with a history of serious aggression towards humans and/or extreme aggression towards other cats and cats with concomitant medical conditions that may interfere with evaluation of effectiveness were excluded. No treatments with facial pheromones or psychoactive nutraceuticals in the 4 weeks prior to day 0 (D0) were allowed for any of the cats in the study. Households with cats receiving any psychotropic medications such as tricyclic antidepressants, selective serotonin reuptake inhibitor or benzodiazepines in the 3 months prior to D0 were likewise excluded. Behavioral modification beyond the instructions provided for handling aggressive events was not permitted. Both the treatment and placebo groups were provided the same recommendations.

Randomization was based on order of attendance at the enrollment meeting; households were assigned to their respective treatment groups (ie, either test article or placebo) according to a simple, blocked, randomization list provided by the study statistician. No stratification factors were utilized. The test materials were provided by Ceva Santé Animale. Each cat owner was provided a single plastic bag containing two identical diffusers that were to be plugged in the home for a 28-day treatment period. Each diffuser was labeled with a unique identification number consisting of two digits according to the randomization list, followed by the letter A or B, indicating pairs of identical treatment vials. The investigator and all support personal at the enrollment center were completely masked to treatment identity and treatment groups. Households were provided with the test articles at the enrollment meeting but were instructed not to install them for 1 week.

During the enrollment meeting, training on the methods of behavioral evaluation to be used in the study was provided; ie, the use of daily diaries and of a scale specifically designed to assess intercat aggressive interactions. Owners were to use the same assessment tools throughout the study. Participants were also provided directions for handling aggressive events (see Appendix 1 in the supplementary material), including classical conditioning, redirection by positive reinforcement and not punishing or startling for aggressive displays. Additionally, owners were given approximately 90 mins of education on the interpretation of cat behavior, including videos of representative intercat interactions to help owners accurately interpret their cats’ behavior over the course of the study. Owners were trained by the behaviorist through description and video illustration to be able to better distinguish between affiliative intercat behaviors, neutral and passive, and overt aggressive behaviors. Examples of positive intercat interactions included nose touching, allogrooming, co-sleeping with close physical contact and tail-up greetings.

**Figure 2:**
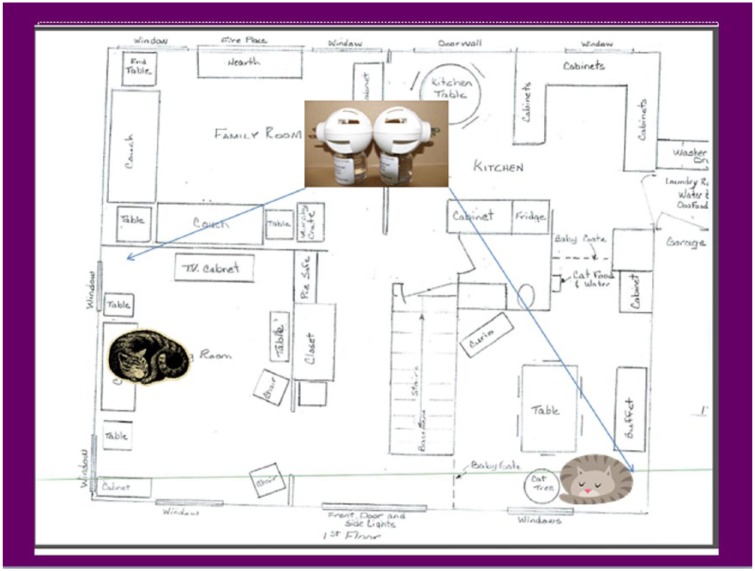
Typical household diagram with cats’ preferred resting areas and diffuser placement

Detailed instructions were provided regarding the use of the diffusers. Each owner drew a diagram of their home’s floor plan and the areas in which the cats spent their time (see example in Figure 2). The optimal location for each diffuser was selected by the investigator based on the primary resting location for involved cats, availability of appropriate electrical outlets and avoidance of open windows. Diffusers were plugged in from D0–D28. Owners were asked to check the warmth and liquid volume in each diffuser weekly and this was confirmed by weekly telephone interview. To assess any potential post-treatment effect of the pheromone or regression following withdrawal, assessments for each household were continued for two additional weeks. Each household’s participation was complete upon conclusion of the day 42 (D42) evaluation.

Two types of assessment methods were used in the study: daily diary entries (from D–7 to D42) and the weekly Oakland Feline Social Interaction Scale (OFSIS) questionnaire (see Appendix 2 in the supplementary material). Daily dairy entries consisted of answering two questions pertaining to the occurrence of aggressive events or fights between the cats in the household, whether they had aggressive encounters or fought that day, and, if so, how many times aggressive behaviors or fighting were observed. Owners were instructed to have the same person complete the diary throughout the study, though input and observations from all family members were allowed. Owners were asked to fax or email diaries weekly to the investigator.

In addition to the daily diary, a weekly questionnaire was used to gain more specific data on the types of aggressive interactions in the home. Beginning on D–7 at the enrollment meeting, then weekly thereafter, owners were asked to describe their cats’ responses to housemate cats and consider all interactions, which they could recall for the previous week. Over the course of the study, owners were instructed not to change their typical routines, but rather assess the level of aggressive interactions based upon their typical level of observation.

The weekly questionnaire used was designated the Oakland Feline Social Interaction Scale (OFSIS)–Aggression and was developed by the investigator specifically for this study based on common owner-reported signs of conflict and on a literature review. The scale was used to provide a quantifiable measure of the frequency and intensity of 12 intercat interactions reflecting conflict between cats. Development of this scale was inspired by the Lincoln Sound-sensitivity Scale, which utilizes a quantifiable measure of the frequency and intensity of signs of noise-related fears.^19^ OFSIS included incidence (yes/no), frequency (how often observed) and intensity (magnitude of response) for each behavior relating to level of aggression between housemate cats. The primary outcome measure for each household was the OFSIS–Aggression score, which was calculated using the following formula: OFSIS aggression score Dx = Σ_(i=1– 12)_(frequency[Q_i_]*intensity[Q_i_]) where, Dx = D–7, D0, D7, D14, D21, D28, D35 and D42, and *Q_i_* = questions 1–12 of the OFSIS questionnaire (first section; see Appendix 2 in supplementary material).

The potential OFSIS–Aggression scores for each household ranged from 0 (no aggressive behaviors at all recorded on the scale) to 360 (each parameter was scored maximally). As the OFSIS was to serve as an assessment of intercat aggression across all cats in the home, a single score for each question applied to the home, not to a specific cat, and each score was to be made according to the most extreme manifestation among their household cats. The 12 behaviors described in the OFSIS questionnaire (see Appendix 2 in the supplementary material) could further be classified by the predominant offensive or fearful manifestations of conflict-related behaviors – offensive (1, 2, 3, 7, 10, 11) and fear (4, 5, 6, 8, 9, 12) – which could be displayed by any cats in the home. To further illustrate affiliative behaviors, the weekly questionnaires also included questions on harmony, which represented how well the cats were getting along with each other and family members.

OFSIS questionnaires were completed in person at the enrollment meeting. OFSIS assessments and the daily diary were returned to the investigator on D0, D7, D14, D21 and D28. Any concerns were clarified during the telephone interview. On D28, owners were also asked ‘Generally, do you find your cats are getting along better?’. A trained technician conducted the phone interviews and recorded a yes/no polar conclusion. Upon conclusion of the D28 telephone interview, owners were instructed to remove the diffusers. To assess any potential post-treatment effect of the pheromone, daily diaries were maintained and two additional weekly OFSIS telephone interviews were conducted on D35 and D42.

### Statistical analysis

SAS 9.3 software (SAS Institute) was used for the statistical analyses. Results are expressed as mean ± SD or n (%). Tests were two-sided and as this was a pilot study the significance threshold was set at 0.10. The experimental unit was the household and the sample size was 45 enrolled households. Baseline characteristics were compared between the two treatment groups. When a clinical difference was found, the difference was tested (Fisher’s exact test). The main analysis was conducted on repeated measures from D0–D28. A generalized linear mixed model (GLIMMIX) for repeated measures was built with comparing the proprietary cat-appeasing pheromone product vs placebo, assessment points, interaction study pheromone by assessment point and baseline as fixed factors. As the group by assessment point interaction was not significant, the interaction was skipped from further models.

**Figure 3:**
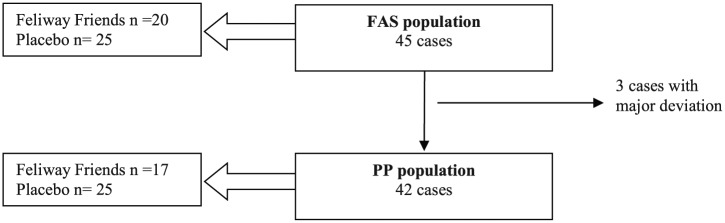
Definition of the analyzed populations. FAS = full analysis set; PP = per protocol

The analyses were performed for both the full analysis set (FAS) and the per protocol (PP) set, with the analysis of the PP population being used in a robustness purpose (Figure 3). Owing to the very low number of cases with a major deviation, the other parameters were analyzed on the FAS population only. As the overall results of the FAS and the PP populations were similar, herein only results from the FAS population are presented. The impact of the number of cats in the household was analyzed in a mixed model with study pheromone and assessment point as fixed factors. Random factors were the number of cats in the household and interaction between the number of cats and group. As this interaction was found to be non-significant, it was removed from the model, which only included treatment group, baseline values and assessment date. In addition, the evolution from D0–D42 was compared between study pheromone using a mixed model for repeated measures.

## Results

Of the 45 households that provided informed consent, 20 were enrolled in the treatment group and 25 in the placebo group (corresponding to the FAS). Forty-two households completed the study: 17 in the treatment group and 25 in the placebo group (corresponding to the PP population). A simple randomization list was utilized, which was balanced on blocks of cases with an anticipated total of 60 cases, but enrollment was ceased at 45 subjects, which resulted in uneven groups. There were three cases of major deviations and all were in the treatment group: one family found and introduced a kitten into the household, one household did not comply with required follow-up and one owner elected to discontinue participation when the aggression resolved but the cat that had previously been reclusive began toileting on household items. In all three deviations, the level of conflict was reported to be improving. No treatment-related adverse events were reported. At D0, there were no differences between the two treatment groups (whether considering FAS or PP populations), except for the number of cats in each household. There were more two-cat households and fewer four- and five-cat households in the pheromone-treated group than in the placebo group (Table 1). The difference in number of cats per household was significant in the FAS population (*P* = 0.04) while trending similarly in the PP population (*P* = 0.09).

**Table 1:** Number of cases per treatment group and number of cats in the household according to treatment group in full analysis set (FAS) and per protocol (PP) populations

	FAS population (n = 45)		PP population (n = 42)	
	Feliway Friends (n = 20)	Placebo (n = 25)	Feliway Friends (n = 17)	Placebo (n = 25)
Two cats	11 (55.0)	5 (20.0)	9 (52.9)	5 (20.0)
Three cats	7 (35.0)	9 (36.0)	6 (35.3)	9 (36.0)
Four cats	1 (5.0)	7 (28.0)	1 (5.9)	7 (28.0)
Five cats	1 (5.0)	4 (16.0)	1 (5.9)	4 (16.0)
	*P* = 0.0409		*P* = 0.0883	

Data are n (%). *P* values correspond to Fisher’s exact test, demonstrating the impact on the number of cats in the household

**Figure 4:**
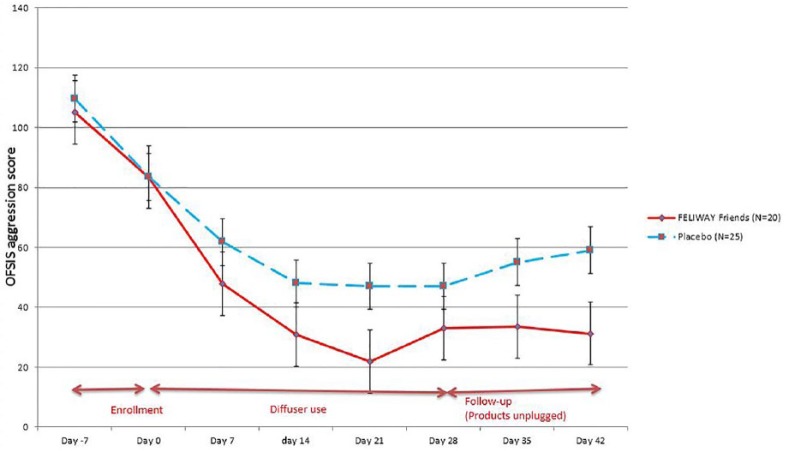
Evolution of Oakland Feline Social Interaction Scale (OFSIS)–Aggression score in the full analysis set population (plot means with SE bars) illustrating the similarity at baseline (mean ± SE) and the difference between groups over the full study period (*P* = 0.0431)

The resulting OFSIS–Aggression scores (possible 0–360) are described in Table 2 and Figure 4. Baseline values were highly correlated between groups on both D–7 and D0. During the enrollment period (D–7 to D0), the OFSIS–Aggression score decreased similarly in both groups. During the treatment period (D0–D28), the OFSIS–Aggression score continued to decrease in both groups, but the decrease was greater in the appeasing pheromone group than in the placebo group. Separation of the group means by treatment was apparent by D7, reaching a distinguishable improvement by D14 (*P* = 0.0833). Mean OFSIS scores reached the highest difference and were significantly different by group on D21 (*P* = 0.0308; Table 2, Figure 4). Diffusers were unplugged on D28 and each household was followed for an additional 2 weeks. Differences between treatment group means persisted during this post-treatment observation period with the treatment group OFSIS scores comparatively stable, while the placebo group mean slowly began to regress (showing worsening conflict). Statistically, there was a trend at D35 (*P* = 0.12) and significance at D42 (*P* = 0.0357). When a repeated-measures analysis was performed on all treatment points collectively from D0–D42 the difference between treatment and placebo was statistically significant (*P* = 0.0431).

**Table 2:** Evolution of Oakland Feline Social Interaction Scale–Aggression score according to treatment group in the full analysis set population

	Feliway Friends (n = 20)	Placebo (n = 25)	*P* value
D–7	105.1 ± 49.6	109.6 ± 42.7	0.74
D0	83.4 ± 46.4	83.5 ± 41.4	0.99
D7	47.8 ± 27.5	61.8 ± 38.5	0.18
D14	30.8 ± 20.7	48.0 ± 41.6	0.0833
D21	21.8 ± 16.7	47.0 ± 37.1	0.0308
D28	33.2 ± 39.8	47.0 ± 48.6	0.34
D35	32.5 ± 33.2	55.0 ± 52.4	0.12
D42	31.2 ± 33.3	59.0 ± 44.8	0.0357

Data are mean ± SD

At inclusion (D–7) all 12 aggressive behaviors (Table 3) were represented in all included households, with 8/12 aggressive behaviors described in the OFSIS questionnaire (staring, stalking, chasing, fleeing, crouching, hissing/growling/spitting, tail twitching/lashing and blocking) displayed in more than 75% of the homes. An overview of descriptive information on the enrolled cats and households is provided in Table 4.

**Table 3:** Reported incidence of aggressive behaviors described in the Oakland Feline Social Interaction Scale questionnaire per households at day 0 (D0)

Intercat interactions reflecting conflict	Reported per households at D0
Staring	44 (97.8)
Fleeing	40 (88.9)
Hissing/growling	39 (86.7)
Chasing other cats	39 (86.7)
Stalking	37 (82.2)
Crouching	37 (82.2)
Twitching tail	36 (80.0)
Blocking	27 (60.0)
Shaking	20 (44.4)
Screaming	20 (44.4)
Remaining hidden	16 (35.6)
Biting	11 (24.4)

Data are n (%)

**Table 4:** Overview of descriptive demographic information on the enrolled cats and households

Subjects	
Number of households	45
Total cats in study (n)	137
Average cats/household (n)	3.0
Average age (years)	6.5
Age range	8 months to 16 years
Female (neutered)	76 (55)
Male (neutered)	61 (45)
Mixed breed	123 (90)
Purebred	14 (10)
Cats per household	
2	16 (36)
3	16 (36)
4	8 (18)
5	5 (11)
Other household pets	
None	21 (47)
Included dogs	19 (42)
Included other pets	7 (16)
Size of home (ft^2^ )	
<1000	6 (13)
1000–1499	16 (36)
1500–1999	11 (24)
2000–2499	4 (9)
2500–2999	3 (7)
⩾3000	5 (11)
Source	
Acquired from friend/family	25 (18)
Shelter/rescue organization	44 (32)
Found/stray	42 (31)
Did not answer/unknown	14 (10)
Breeder	7 (5)
From veterinarian	4 (3)
Born in household	1 (1)
Declaw status: by home	
Include one or more declawed cat	27 (60)
Include no declawed cats	18 (40)
Declaw status: by cat	
Not declawed	72 (53)
Declawed	65 (47)
Declawed front only	48 (35)
Declawed all four	17 (12)
Aggression: duration	
Range	17 days to 11.5 years
Average	901 days (30 months)
Median	594 days (19.8 months)
Aggression: nature of onset*	
Noted upon initial introduction	15 (33)
Noted after the cats had been living together without apparent conflict	28 (62)
Reported cause or worsening in association with a specific event	25 (56)
Gradual onset (indeterminate cause of escalation)	3 (7)
Uncategorized	6 (13)
Owner identified as aggressor or victim^†^	
Aggressor	66 (48)^‡^
Male aggressor	36 (55)
Female aggressor	30 (45)
Declawed aggressor	29 (44)
Non-declawed aggressor	37 (56)
Victim	52 (38)^‡^
Male victim	17 (33)
Female victim	35 (67)
Declawed victim	31 (60)
Non-declawed victim	21 (40)
Both aggressor and victim	16 (12)^‡^
Male aggressor and victim	8 (50)
Female aggressor and victim	8 (50)
Indifferent/not involved	21 (15)^‡^
Not sure or did not answer	18 (13)^‡^

Data are n (%) unless otherwise indicated

* Some owners gave multiple answers, suggesting behaviors as different cats were introduced

† Cats classified as aggressor, victim, indifferent, not sure. Multiple labels provided

‡ Of all cats

Feline harmonious and affiliative behaviors may be subtle and poorly identified by owners.

Questions about the affiliative interactions within the housemate cats were included. Of the eight affiliative behaviors included in the OFSIS–Harmony questionnaire (Appendix 2 in the supplementary material), three (nose-touching, sleeping in the same room at the same time, licking around the head or neck of a housemate cat) were displayed by >60% of cases, and three others (greeting family members, tail-up posture during home-coming greeting, sleeping in moderate contact) by around 50% of cases. The other two (rubbing on a person during home-coming greeting, snuggling or sleeping with a seated person) were generally displayed by <40% of cases. Using the sum of the yes answers to the eight harmony questions did not show any difference between the two groups. The OFSIS–Harmony score was similar in both groups at D–7 and D0 and then only slightly higher in the appeasing pheromone group throughout the study period (ranging from 11.1–13.9 in the appeasing pheromone group and from 11.2–12.4 in the placebo group). From D7 onwards it was always higher in the pheromone-treated group than in the placebo group, but the difference was not significant. Moreover, the overlap of SE values confirmed there was no statistically significant difference (*P* >0.05) in OFSIS–Harmony scores between the treated and the placebo groups.

Ultimately, owner perception of satisfaction with the product and response to treatment was asked at D42. Enrollment in the study was based on owner perception of conflict between housemate cats and thus the query ‘Generally, do you find that your cats are getting along better?’ was used to assess the owner perception of amelioration of that conflict. In the cat-appeasing pheromone group 84.2% vs 64% in the placebo group reported that they perceived their cats were getting along better (*P* = 0.14).

## Discussion

Demographic data (Table 4) were reviewed for patterns as compared with general populations but considered too few to assess for treatment effect. In this study, the average number of cats per home was 3.0 and 13/45 homes in this study (29%) had four or five cats. Of the 137 cats, 55.4% were female. Most were mixed-breed cats, but 10% were purebred cats. Owners identified 66 cats as the aggressor and only a slightly higher proportion of those were males: (30 female [45%] and 36 male [55%]), whereas twice as many females as males were considered a victim (67% vs 32%).

Owing to the unequal number of cats per households in both groups, and as the randomization was not stratified according to the number of cats, this criteria was included as a covariate in the statistical mixed model. The interaction between number of cats per home and treatment group was tested and found to be non-significant (*P* = 0.9). This meant that the unequal number of cats per home did not have an impact on the results. Consequently, this interaction was removed from the statistical model, which only included treatment group, baseline values and assessment date.

Variables such as the square footage of a home, the number of floors of a home and the areas to which cats have access are not easily controlled and may be useful data to collect but are difficult to interpret in a study of this size. According to 2013 US Census information the average home in the USA was reported to be 1500 square feet, with homes in the Midwest average ranging from 1615–2265 square feet, depending on the year built (1973–2010).^35,36^ In this study, 82% of the homes were <2499 square feet and 49% of homes were <1499 square feet (see Table 4), consistent with the common assertion that less available territory is consistent with maintenance of feline conflict. The availability and distribution of cats’ resources in the home is a key element of intercat relationship factors, but they are difficult to assess in this type of study. Home size is also consistent with package directions that at least two diffusers should be provided to cover the size of these homes.

Conflict between housemate cats is generally considered to have a poor prognosis for successful alleviation of the signs of conflict, occurrence of reconciliation and development of affiliative bonds between housemate cats. To our knowledge, this is the first randomized and placebo-controlled study to evaluate a treatment intervention in households with intercat aggression. In this study a synthetic analog of an appeasing pheromone was hypothesized to influence social bonding based on the natural occurrence of high levels of cat-appeasing pheromones delivered from the queen prior to and simultaneous to the occurrence of the socialization window. The primary investigator (TLD) speculated that there may be a natural and causal relationship between the presence of appeasing pheromones and the enhancement of social interactions. Thus, the authors speculate that the beneficial effect on social interactions is achieved by similar mechanisms as during the natural pliable socialization window.

As intercat aggression has been insufficiently studied, the primary investigator (TLD) was required to develop a useful metric to assess the frequency and severity of aggression within the home. The OFSIS captures the frequency and intensity of 12 behaviors reflecting intercat aggression, and the total OFSIS score was formed by the summation of the 12 scores. Thus, the OFSIS score could theoretically range from 0 for cats that exhibited none of the listed aggressive behaviors to 360 for homes where cats exhibited the entire constellation of listed behaviors with maximum frequency and intensity.

The predominant limitation of the OFSIS is that it relies entirely upon owner interpretation, making validation complicated. Future studies should include validation by either a test–retest reliability or an inter-rater reliability assessment. Low-level or passive aggression is likely to be under-reported or under-represented. Moderate or extreme overt aggression would be clear but could result in a higher rate of relinquishment, and thus cats with extremely high OFSIS scores are also potentially under-represented. The owner’s perception of conflict is actually a pivotal factor as the owner may resolve to relinquish a cat that poses the greatest risk to a cat’s welfare and wellbeing. Although the volunteers did record the number of fights and aggressive encounters per day, these parameters were not useful in analysis as these did not successfully quantify the severity. Owner interpretation of ‘fights’ did vary as the aggressive encounters ranged from passive staring to fights. Some owners reported finding tufts of fur. Thus, there were differences between the initial weekly number of fights and weekly number of aggressive encounters in both groups (at D0), making this parameter less relevant for analysis, whereas the OFSIS scores, reflecting globally all aspects of the conflict, were similar at baseline.

Analysis of the scale’s component questions was performed to reveal if simplification of the scale was appropriate and it was determined the scale was most useful in its entirety. When the 12 questions were assessed separately for frequency and intensity the results were not more discriminating: all of the questions were represented at inclusion (Table 3), and all 12 decreased in frequency and intensity throughout the study period, regardless of their initial incidence, differently between the two groups. For example, ‘fleeing from housemate cats’, a very frequent sign reported in 90% of appeasing pheromone homes and 88% of placebo homes at D0, was only reported in 55% and 76%, respectively, at D21, whereas ‘screaming’, only reported by 40% of appeasing pheromone homes and 48% of placebo homes at D0, decreased up to 10% and 24%, respectively, at D21. While the manifestation of conflict may vary between different households, the composite design of the scale accounted for this expected variability by quantifying each behavior associated with intercat conflict (eg, staring vs stalking vs biting) while simultaneously qualifying the intensity. The scale was intended to capture changes in these parameters over time and, potentially, in response to treatment, realizing that certain aggressive behaviors may change in intensity or frequency or both. The aggressive behavior may be considered as a matrix of frequency vs intensity of signs of conflict and provided a range of options for owner reporting.

As a practical matter, the households in the study clustered around the lower half of the scale, indicating that cats tended to exhibit several behaviors, but not all behaviors, and that the most extreme cases were not represented, partially because they were purposely excluded from the study for safety and welfare reasons. As feline interactions change there is also a change in the manifestation of signs as some cats were hiding at the onset of the study and thus rarely present to participate in more passive behaviors such as staring or blocking.

The strength of the above approach is that it captured a variety of data across the multidimensional nature of aggression. A potential weakness, however, is that the scale gives equal weight to each possible behavior. In raw numerical terms, a household with cats that have frequent episodes of intense fighting may be given a score equal to another home where the cats do not fight at all but have frequent episodes of intense staring or stalking. Both conditions represent an adverse impact on the cats’ welfare. Whether these situations are, in fact, ‘behaviorally equivalent’ is an open question, but the scale assumes they are. The OFSIS should be qualified by a test and retest statistical approach as validation by any ‘gold-standard’ diagnosis is not possible and all diagnoses will rely on the clinician’s interpretation of owner descriptions.

The households enrolled in the study all had intercat aggression evaluated on D–7; however, most experienced a drop in the OFSIS score prior to D0, in the absence of any treatment. It is possible the owners over-reported the level of aggression that had occurred in the 7 days prior. Though repeatedly reminded to consider only the immediate prior 7 day period, owners were motivated to participate and concerned about their cats’ welfare regarding a problem that had existed, in some cases, for months or years. Given the consistency of this observation across the households, the authors attribute this drop – in the absence of contrary evidence – to the effects of the training session with the behaviorist and primary investigator on D–7. Merely understanding the reasons why cats may act out with aggression may have reduced the owner’s perception of the signs of conflict. Further, changing the strategies from a punitive-based to a positive reinforcement-based approach may have immediately reduced stress for the owners and the cats.

It seems instinctive for owners to wish to intervene in whatever manner is effective to stop an aggressive event between cats. Even many veterinarians have recommended the use of distractors such as spray bottles, and loud noises in order to stop an aggressive event. While these interventions may result in a temporary victory, it is often pyrrhic and short lived. Cats typically resume their aggressive behavior when what they perceive as the greater threat (eg, the water bottle or the noise) is dissipated. Aggressive behaviors should be redirected whenever feasible by using positive reinforcement and cues. As owners were instructed to implement such changes, the decrease in OFSIS score prior to D0 seems attributable to the change in intervention tactics.

An interesting finding was the improvement of the OFSIS–Aggression score before treatment, D–7 to D0, in both groups (Figure 4). During the initial training meeting, at least one representative from each household was given instruction on cat behavior and how to appropriately and effectively intervene when intercat aggression arose. Punishment was discouraged (eg, spraying cats with water) and positive redirection strategies (eg, food) were suggested. This change in management at home may be sufficient to explain the drop in mean scores between D–7 and D0, irrespective of group. These management strategies, while confounding, were undertaken in consideration for the welfare of the cats and because most interventions that reduce anxiety may be less effective if punishment is imposed. This may have contributed to a relevant portion of the improvement seen in both groups throughout the study.

However, unexpectedly after the treatment period, the OFSIS score in the treatment group remained stable while the aggression score in the placebo group began to rise (Figure 4). The authors postulate that the pheromone treatment enhanced the affected cats’ learning ability and initiated a new balance, so-called reconciliation, in their social interactions, resulting in a more persistent behavioral change after the treatment was withdrawn on D28 (Figure 4). While it is interesting that cats in pheromone-treated households continued to improve (as demonstrated by the OFSIS score) during the brief 2 week post-treatment period, this short period is insufficient to draw a long-term conclusion. Perhaps long-term treatment with pheromones may be beneficial but may not always be required, as cats that learn how to ‘get along’ may retain that ability, but this is beyond the scope of this study. It is uncertain whether any post-treatment effect would be reduced by changes in the home such as introduction or removal of animals, a member of the family moving in or out, or movement of the entire household; all of which are known to affect feline behavior and, potentially, intercat aggression. Future studies should examine a longer post-treatment period.

Difference between groups was found to be statistically significant over the full study period (D0–D42). The highest differences between groups occurred at D14, D21 and D42. The most important clinical effect for the pheromone treatment seemed to occur within the first 14–21 days. This time interval allows for saturation of the environment by the diffusers, opportunity for the cats to have been sufficiently exposed to the pheromones, modulation of each cat’s perception and the impact of learning/anticipation there may be a change in social interactions.

While the OFSIS–Aggression proved discriminatory for measuring conflict, the assessment of affiliative interactions by the OFSIS–Harmony, as designed, was less insightful. The harmony questions were complicated for clients to answer in multi-cat households, which included not only populations of aggressive dyads, but also, in some households, affiliative dyads. The OFSIS–Harmony questions were designed to ask about all cats in the home and not specific dyads. The question format (eg, ‘*any* of your cats’ and ‘did *all* of your cats’) proved very difficult for owners to answer. Further, some behaviors, such as sleeping in the same room together, occurred commonly in households but may not be a good measure of affiliative interactions in cats as some cats did not have another option for resting areas. The authors believe measuring an increase in harmonious interactions along with a concomitant decrease in intercat aggression would be useful in evaluating intercat behaviors, but the OFSIS–Harmony questions should be revised in future versions.

Evaluation of the composition of homes with cats that do not get along is intriguing, but interpretation has limitations as there are correlations that may not be causal. Further, the population of cats that do not get along is plagued by a culling process: cats, both the aggressors and the victims, in highly volatile homes may be removed by relinquishment, euthanasia or simply by escaping/running away. Factors that influence retention rather than relinquishment of a cat do have a dramatic impact on human decisions: some cats were inherited from a loved one, whereas others were acquired from a breeder, and these factors may impact strongly on retention, despite severe or persistent conflict. Sixty-two percent of the cats were noted to be aggressive after the cats had been living together without apparent conflict, which suggests that either owners are unaware of the signs of low-level conflict, or cats may become conflicted and not reconcile spontaneously.

A surprising finding in this study (Table 4) was the unexpectedly high percentage of declawed cats: 47.4% vs 24.4% declawed cats in the overall cat population of the USA.^11^ Among the aggressors, 44% (29/66) were declawed, whereas 56% (37/66) were not declawed. Among the victims, 60% (31/52) were declawed, whereas 40% (21/52) were non-declawed. While this trends towards an adverse effect of declawing on feline social relationships, these findings were not statistically significant. The investigator has completed two subsequent and similar studies on intercat conflicts, which are beyond the scope of this paper. An analysis of these unpublished data showed that of the 757 cats from 246 households 33.9% (n = 257) were declawed, which further supports the suggestion in the present study that there may be a correlation between declaw status and ongoing conflict between housemate cats.

The investigator speculates that declaw status of either the aggressor or the victims may have an impact on ongoing aggression. If sufficient defense is not possible by the declawed cat, the threat of dangerous weapons is described as a primary ethological reason for the complex facial communications shared by all domestic and wild felids.^9^ This finding is confounded by multiple cats nested within households and it is unclear if cats were declawed as a result of the ongoing conflict or if the declawing preceded the onset of aggression. Cats may be declawed much more commonly in the USA than in other parts of the world.^11^ Regardless, the high percentage of cats that are declawed and in homes with intercat aggression is an interesting finding that warrants future investigation.

## Conclusions

In this study, treatment with a proprietary cat-appeasing pheromone diffuser for 4 weeks showed a beneficial effect in the management of feline aggression in multi-cat households. During the study, when cat owners were educated by a board-certified veterinary behaviorist about feline behavior, provided instruction on handling aggressive events and discouraged from punishing cats (eg, squirt guns or other startle methods), the level of conflict began to decrease even prior to implementation of the treatment. Pheromones may be useful as a component of a complete behavior modification program.^8,15,16,21^ Considering the duration of aggression in these homes averaged 901 days (median 594 days), the ability of the treatment to have an impact on long-term aggression in only 4 weeks is promising. The OFSIS–Aggression score is a useful tool in the evaluation and monitoring of aggression between housemate cats in a field setting.

Demographic data suggest that declawing may be associated with increased conflict between housemate cats and that both sexes were almost as likely to be the aggressor, though females were more often the victims. Furthermore, educating owners regarding the meaning of feline postures, charting progress by means of the OFSIS, ceasing of punishment, implementing classical conditioning and use of an appeasing pheromone that enhances social interactions together provide a complete professional program for assisting owners and their cats.

## Supplemental Material

Appendix_1._INSTRUCTIONS_FOR_HANDLING_AGGRESSIVE_EVENTS – Supplemental material for Evaluation of the efficacy of an appeasing pheromone diffuser product vs placebo for management of feline aggression in multi-cat households: a pilot study Click here for additional data file. Supplemental material, Appendix_1._INSTRUCTIONS_FOR_HANDLING_AGGRESSIVE_EVENTS for Evaluation of the efficacy of an appeasing pheromone diffuser product vs placebo for management of feline aggression in multi-cat households: a pilot study by Theresa L DePorter, David L Bledsoe, Alexandra Beck and Elodie Ollivier in Journal of Feline Medicine and Surgery

## Supplemental Material

Appendix_2._OFSIS – Supplemental material for Evaluation of the efficacy of an appeasing pheromone diffuser product vs placebo for management of feline aggression in multi-cat households: a pilot study Click here for additional data file. Supplemental material, Appendix_2._OFSIS for Evaluation of the efficacy of an appeasing pheromone diffuser product vs placebo for management of feline aggression in multi-cat households: a pilot study by Theresa L DePorter, David L Bledsoe, Alexandra Beck and Elodie Ollivier in Journal of Feline Medicine and Surgery
