# Supplementary material for: Evaluation of the efficacy of an appeasing pheromone diffuser product vs placebo for management of feline aggression in multi-cat households: a pilot study
Source: J Feline Med Surg. 2018 May 14;21(4):293–305. doi: 10.1177/1098612X18774437 (PMC6435919; doi:10.1177/1098612X18774437)
Supplement: Appendix_1._INSTRUCTIONS_FOR_HANDLING_AGGRESSIVE_EVENTS – Supplemental material for Evaluation of the efficacy of an appeasing pheromone diffuser product vs placebo for management of feline aggression in multi-cat households: a pilot study [file Appendix_1._INSTRUCTIONS_FOR_HANDLING_AGGRESSIVE_EVENTS.docx]

**INSTRUCTIONS FOR HANDLING AGGRESSIVE EVENTS**

1. Do not punish cats for aggressive behaviors.
2. Do not reprimand, yell, or startle cats for aggressive behaviors.
3. Do not spray cats with water, squirt with air or throw items at cats for aggressive behaviors.
4. Be calm. Do not yell or panic. Be quiet and move slowly.
5. If the encounter is mild or minor in intensity, your cats may be best left to “work it out on their own”. This does not mean let your cats fight it out!!! (ie. if cats are staring or blocking – you may not need to intervene.)
6. Play wrestling is not the same as fighting or aggressive events. During play wrestling cats are silent. There is more physical contact including holding a paw on the other cat, wrapping body around and ‘bunny kicking” with hind legs. Both cats take turns on their backs. Either cat may run or chase. Either cat may wiggle or pounce.
7. If you are concerned about an aggressive encounter, you may encourage either cat to move away by using a sweet, gentle tone of voice. You may coax your cat by inviting for food, treats or toys. You may make noises that you know cause your cats to happily investigate (ie go to the kitchen and open a can of tuna).
8. If necessary, you may intervene. Only intervene if the risk for injury seems high or the cats have continued in a stand-off for >20 seconds.
9. Do not pick up a cat that is aroused. The risk for accidental aggression to people is very high when the cat is aroused.
10. Acceptable interventions: block your cat’s view of each other with an available object (not your body!). Use a sofa cushion, poster board sized paper or calmly position any available object between them. Do not threaten or push either cat. Do not shoo or chase your cats away. Give them time to move off slowly on their own.
11. You may gently drop a blanket over one cat to obscure the view of each other. This is not a “capture” technique – this is intended to diffuse the aggression by obscuring their view and creating a hiding place. Be especially careful to avoid touching a cat that is highly aroused and “explosive”.
12. If your cats are actually fighting: which includes extreme vocalizing and active biting/scratching which continues you may startle, stomp, yell or to end the incident safely. Avoid doing this since while this may end the event, this damages the cats’ relationship further and makes future aggressions more likely and more difficult to resolve.
13. Do not reprimand your cats afterward. Do not have a discussion with them explaining why they should be nice.
14. Ok to confine a cat after an aggressive incident. Close doors to separate cats. Do not corral, chase or capture a cat after an incident. Ok to let the victim cat hide in a bedroom with the door shut. It is not necessary to restrict the “bad” cat.
15. Be sure to note the incident in the daily journals.
