# Supplementary material for: Evaluation of the efficacy of an appeasing pheromone diffuser product vs placebo for management of feline aggression in multi-cat households: a pilot study
Source: J Feline Med Surg. 2018 May 14;21(4):293–305. doi: 10.1177/1098612X18774437 (PMC6435919; doi:10.1177/1098612X18774437)
Supplement: Appendix_2._OFSIS – Supplemental material for Evaluation of the efficacy of an appeasing pheromone diffuser product vs placebo for management of feline aggression in multi-cat households: a pilot study [file Appendix_2._OFSIS.doc]

**The Oakland Feline Social Interactions Scale** ID: _____Name: _____________________

Date: _________

D-7 D0 D7 D14

D21 D28 D35 D42

Please describe your cats’ responses to housemate cats. This is your impression of the behaviors of all of your cats. It is not necessary to follow your cats but please note your cats’ interactions. These questions refer to ANY cat in your home which may display these behaviors. Consider all of your cats when you consider responses. Score according to the most extreme manifestation amongst your household cats.

- The first section includes incidence (yes/no), frequency (how often you observe) and intensity (magnitude of response you observe) for each behavior relating to level of aggression between housemate cats.
- The second section relates to harmony in household and includes incidence (yes/no) and frequency (how often you observe) for each behavior.
- Complete once a week. Please pick one best answer. Please be sure to complete every question.
- Consider your observation of your cats’ behaviors in the *previous* week.
- Please scan and email forms to [**Theresadvm@aol.com**](mailto:Theresadvm@aol.com). Alternatively forms may be sent regular mail or fax.
- These scores are used to reflect your opinion of your cats’ behavior. The same person should complete the form each week. Ok to discuss with all family members as observers.

On average this week, how many hours per day were you or a family member at home with your cats? You don’t have to be continuously observing your cats. How many hours were you present that you would have heard or seen an aggressive encounter or fight?

Pick the one best answer:  21-24 hours/day

17-20 hours/day

13-16 hours/day

9-12 hours/day

5-8 hours/day

0-4 hours/day

**Cats’ interactions / level of aggression**

Considering the last week, describe the incidence (yes/no), frequency (how often you observe) and intensity (magnitude of response you observe) for each behavior:

1. **Did any of your cats stare at each other? No**  **Yes**

If yes, score frequency: Rarely Sometimes Occasionally Most of the time Every time

If yes, score intensity: 1 2 3 4 5 6

Low, minimal extreme

1. **Did any of your cats stalk each other? No**  **Yes**

If yes, score frequency: Rarely Sometimes Occasionally Most of the time Every time

If yes, score intensity: 1 2 3 4 5 6

Low, minimal extreme

1. **Did any of your cats chase other housemate cats? No**  **Yes**

(Reminder: all questions refer to housemate cats. Not unfamiliar cats)

If yes, score frequency: Rarely Sometimes Occasionally Frequently Every time

If yes, score intensity: 1 2 3 4 5 6

Low, minimal extreme

1. **Did any of your cats display fleeing from housemate cats? No**  **Yes**

(Fleeing is to run or move very quickly or hastily)

If yes, score frequency: Rarely Sometimes Occasionally Most of the time Every time

If yes, score intensity: 1 2 3 4 5 6

Low, minimal extreme

1. **Did any of your cats display crouching in response to a housemate cat? No**  **Yes**

(Crouching is a low, tucked body posture)

If yes, score frequency: Rarely Sometimes Occasionally Most of the time Every time

If yes, score intensity: 1 2 3 4 5 6

Low, minimal extreme

1. **Did any of your cats exhibit shaking, shivering, trembling or avoidance in response to a housemate cat? No**  **Yes**

If yes, score frequency: Rarely Sometimes Occasionally Most of the time Every time

If yes, score intensity: 1 2 3 4 5 6

Low, minimal extreme

1. **Did any of your cats bite or attempt to bite a housemate cat? No**  **Yes**

If yes, score frequency: Rarely Sometimes Occasionally Most of the time Every time

If yes, score intensity: 1 2 3 4 5 6

Low, minimal extreme

1. **Did any of your cats hiss, growl or spit at each other? No**  **Yes**

If yes, score frequency: Rarely Sometimes Occasionally Most of the time Every time

If yes, score intensity: 1 2 3 4 5 6

Low, minimal extreme

1. **Did any of your cats wail or scream at each other? No**  **Yes**

If yes, score frequency: Rarely Sometimes Occasionally Most of the time Every time

If yes, score intensity: 1 2 3 4 5 6

Low, minimal extreme

1. **Did you observe any of your cats twitching or lashing their tail in response each other?**

**No**  **Yes**

If yes, score frequency: Rarely Sometimes Occasionally Most of the time Every time

If yes, score intensity: 1 2 3 4 5 6

Low, minimal extreme

1. **Did any of your cats block each other? No**  **Yes**

(Blocking is a passive way to prevent another cat’s passage to desired resource)

If yes, score frequency: Rarely Sometimes Occasionally Most of the time Every time

If yes, score intensity: 1 2 3 4 5 6

Low, minimal extreme

1. **Did any of your** **cats remain hidden at times you expected them to come to you?**

**No**  **Yes**

If yes, score frequency: Rarely Sometimes Occasionally Most of the time Every time

If yes, score intensity: 1 2 3 4 5 6

Low, minimal extreme

Considering all interactions this week, how would you rate the aggression displayed by your household cats?

Pick the **one** answer that best describes aggression displayed by your household cats:

1 2 3 4 5 6 7 8 9 10

*1=*barely noticeable *10=*Aggression could not be worse

******************************************************************************************************************

**Harmony in household**

Considering the last week, describe the incidence (yes/no) and frequency (how often you observe) for each behavior:

1. **Did all of your cats come out to greet you when you came home? No**  **Yes**

If yes, score frequency: Rarely Sometimes Occasionally Most of the time Every time

1. **During home-coming greeting, did all of your cats display rubbing on a person (cheek, side and/or back)?** **No**  **Yes**

If yes, score frequency: Rarely Sometimes Occasionally Most of the time Every time

1. **During home-coming greeting, did all of your cats display a tail-up posture?** **No**  **Yes**

If yes, score frequency: Rarely Sometimes Occasionally Most of the time Every time

1. **Did any of your cats display nose–touching to a housemate cat? No**  **Yes**

(Nose-touching is a brief nose-to-nose contact between two cats)

If yes, score frequency: Rarely Sometimes Occasionally Most of the time Every time

1. **Did you observe all of your cats sleeping in the same room at the same time?** **No**  **Yes**

If yes, score frequency: Rarely Sometimes Occasionally Most of the time Every time

1. **Did any of your cats sleep so close together they were in moderate physical contact?** (Moderate physical contact would mean greater than 25% bodies touching) **No**  **Yes**

If yes, score frequency: Rarely Sometimes Occasionally Most of the time Every time

1. **Did any of your cats groom a housemate cat by licking around their head and neck?**

**No**  **Yes**

If yes, score frequency: Rarely Sometimes Occasionally Most of the time Every time

1. **Did all of your cats come to snuggle or sleep with you when you are seated? No**  **Yes**

(Family members seated could include relaxing, reading, working or watching TV)

If yes, score frequency: Rarely Sometimes Occasionally Most of the time Every time

Considering all interactions this week, how would you rate the overall harmony of relationships and quality of friendships between all of the cats in your home:

Pick the **one** answer that best describes the relationship of all cats in your home:

1 2 3 4 5 6 7 8 9 10

*1=*Worst enemies *10=*Best of friends

Considering all interactions between your cats and the family members this week, how would you rate the overall harmony of relationships and quality of friendships between your cats and the family members in your home:

Pick the **one** answer that best describes the relationship of all cats and people in your home:

1 2 3 4 5 6 7 8 9 10

*1=*Aloof, distant *10=*Best of friends

Copyright © 2013 Theresa DePorter DVM - All Rights Reserved
